# *Thuja sutchuenensis* Franch. Essential Oil Ameliorates Atopic Dermatitis Symptoms in Mice by Modulating Skin Microbiota Composition and Reducing Inflammation

**DOI:** 10.3390/microorganisms13122653

**Published:** 2025-11-22

**Authors:** Nana Long, Youwei Zuo, Jian Li, Renxiu Yao, Quan Yang, Hongping Deng

**Affiliations:** 1 Center for Biodiversity Conservation and Utilization, School of Life Sciences, Southwest University, Beibei, Chongqing 400715, China; 2 School of Laboratory Medicine, Chengdu Medical College, Chengdu 610500, China; 3 Chongqing Xuebaoshan National Nature Reserve Management Center, Kaizhou, Chongqing 405400, China

**Keywords:** *Thuja sutchuenensis*, atopic dermatitis, microbial diversity, network pharmacology, JAK1/STAT3 signaling pathway

## Abstract

Atopic dermatitis (AD) is a chronic inflammatory skin disorder characterized by dysregulated immunity, skin barrier dysfunction, and cutaneous microbiome dysbiosis. While current therapies face limitations, *Thuja sutchuenensis* essential oil (TEO) shows promise due to its multi-target potential. We sought to explore the beneficial effects of TEO and delve into its mechanistic actions in a mouse model of AD. We combined network pharmacology with in vivo validation to evaluate the therapeutic efficacy and mechanisms of TEO in an AD model, and confirmed network-predicted targets in an in vitro inflammatory cell model. In AD mice, TEO alleviated pruritus and epidermal hyperplasia, suppressed systemic IL-4/TNF-α and IgE, and partially normalized serum ALB, LDL-C, and HDL-C. Microbial diversity increased after treatment, although potentially pathogenic taxa (*Arthrobacter* sp. and *Corynebacterium mastitidis*) remained enriched. Machine-learning analysis indicated the highest predicted metabolic activity in CK controls, whereas the AD and TEO groups showed elevated pathogenic phenotype scores. Network pharmacology prioritized active compounds [(E)-ligustilide, senkyunolide A, 3-butylisobenzofuran-1(3H)-one, butylated hydroxytoluene, Z-buthlidenephthalide, and β-Myrcene] and core targets (TNF, PTPRC, CCR5, JAK1), implicating T-cell receptor signaling, *Staphylococcus aureus* infection, and STAT3 pathways. Docking and molecular dynamics supported strong, stable binding of major constituents to JAK1, and Western blotting confirmed TEO-mediated inhibition of the JAK1/STAT3 axis. TEO effectively alleviates atopic dermatitis symptoms by modulating immune responses and enhancing microbial diversity. It targets key signaling pathways, such as JAK1/STAT3, highlighting its potential as a therapeutic option for AD.

## 1. Introduction

Atopic dermatitis (AD), a chronic inflammatory skin disorder characterized by immune dysregulation and epidermal barrier dysfunction that affects 15–30% of children and 2–10% of adults worldwide. Clinically, it manifests with severe pruritus, erythema, excoriation, and eczematous lesions [1]. Genetic, environmental factors, skin barrier dysfunction, and immune dysregulation are the primary pathological mechanisms underlying AD [2]. Current therapies, including corticosteroids and calcineurin inhibitors, are limited by side effects and recurrence upon discontinuation [3]. While emollients remain the cornerstone of AD management, the systemic treatment paradigm has evolved significantly in recent years, transitioning from conventional immunosuppressants to targeted biologics (e.g., IL-4/IL-13 inhibitors) [4,5] and, more recently, small-molecule therapies (e.g., JAK inhibitors) [6]. Despite remarkable advances in biologic therapies, contemporary AD management still necessitates a multi-pronged approach targeting four key pathological components: (1) inflammation control (anti-inflammatory agents), (2) pruritus relief (anti-pruritics), (3) prevention of bacterial superinfection (antimicrobials), and (4) epidermal barrier repair (emollients) [7].

Natural products, with multi-target anti-inflammatory properties, have emerged as promising alternatives for AD treatment due to their accessibility, low toxicity, and favorable pharmacological profiles [8,9]. Essential oil is widely used in traditional Chinese medicine owing to its unique hydrophobic and lipophilic properties and enhanced cutaneous permeation [10]. *Thuja* species (*Cupressaceae*), traditionally used in Chinese medicine for skin ailments, liver diseases, bullous bronchitis, and rheumatism produce essential oils containing bioactive terpenoids—notably monoterpenes (α-pinene, β-thujone) with demonstrated anti-inflammatory and antimicrobial properties [11,12]—along with sesquiterpenes that represent characteristic bioactive constituents of *Cupressaceae* plants, responsible for their diverse pharmacological effects [11]. Notably, active ingredient myrcene suppresses NF-κB and MAPKs to limit inflammation responses [13], while linalool modulates Th2-mediated inflammation [14]. As a well-established Th2-dominant disease, AD pathogenesis involves hyperactivated JAK-STAT signaling (particularly JAK1-mediated pathways) in which IL-4 stimulation induces JAK1/JAK3 phosphorylation and subsequent STAT6 nuclear translocation, ultimately driving the expression of Th2-polarized genes in lymphocytes [15]. Previous studies report that the essential oil of *Thuja sutchuenensis* shows antifungal activity against multiple human-pathogenic fungi, and related work has isolated antibacterial sesquiterpenes from its tissues—together indicating pharmacological potential relevant to this study [16]. Nevertheless, the therapeutic potential of TEO for AD remains unexplored, particularly its dual role in immune modulation and as a barrier.

## 2. Materials and Methods

### 2.1. Plant Material

Fresh *Thuja sutchuenensis* Franch. material was obtained in summer 2023 from the artificial cultivation base of *T. sutchuenensis* within the Xuebaoshan National Nature Reserve (Chongqing, China), after three consecutive sunny days. The plant leaves were collected from cultivated plants during dry weather, placed in sterile paper bags, and transported to the laboratory within 24 h. Botanical identity was authenticated by Professor Hongping Deng (Southwest University), and a voucher specimen (No. CQNM 01295) was deposited in the Chongqing Natural History Museum Herbarium (CQNM), Chongqing, China. Fresh leaves of *T. sutchuenensis* (100 g each) were hydrodistilled using the Clevenger apparatus for 4 h to obtain essential oils [17]. A total of 1 mL of essential oil was weighed separately, and the average value was calculated as its density (ρ = 0.8499 g/mL).

### 2.2. Analysis via Gas Chromatography Coupled with Mass Spectrometry

Gas chromatography–mass spectrometry (GC-MS) analysis was performed using an Agilent 5975B mass selective detector coupled with a DB-5MS fused silica capillary column (5% phenyl-methylpolysiloxane, 30 mm × 0.25 mm i.d., 0.25 μm film thickness; Agilent Technologies, Shanghai, China). The injector and interface temperatures were maintained at 250 °C and 290 °C, respectively. The oven temperature was programmed from 70 °C to 290 °C at a rate of 5 °C/min, followed by a 10 min isothermal hold. High-purity helium carrier gas was used at a constant flow rate of 1.0 mL/min. Essential oil samples (10 mg dissolved in 1 mL diethyl ether) were injected in pulsed split mode (1.5 mL/min for 0.5 min, then reduced to 1.0 mL/min for the remaining analysis; split ratio 40:1). The mass spectrometer operated in electron impact mode at 70 eV, scanning the *m*/*z* range of 35–650 at 0.34 s per scan. Compounds were identified by comparing their retention indices (RI, calibrated with C7-C40 n-alkanes) and mass spectra against the Wiley 6, NIST11, MassFinder 2.3, and an in-house MS database (Table A1). TEO was individually emulsified with Tween 80 to obtain a maximum concentration of 10% *v*/*v* (85.0 mg/mL) before administration to the animals.

### 2.3. Animals

C57 bl/6 mice (females, 18–22 g) were purchased from Chengdu Dossy Expereimental Animals Co., Ltd. (Chengdu, China). All animal procedures were performed in accordance with the European Community guidelines (EEC Directive of 1986; 86/609/EEC) and approved by the Animal Ethics Committee of Southwest university (IACUC-20241125-01). All animals were required to undergo institutional quarantine for 7 days prior to use. The mice were housed in an environment with temperature 23 ± 2 °C and humidity 55 ± 10% with a 12 h light/dark cycle, and provided with food and water ad libitum.

#### 2.3.1. Atopic Dermatitis Model

**Figure 1:**
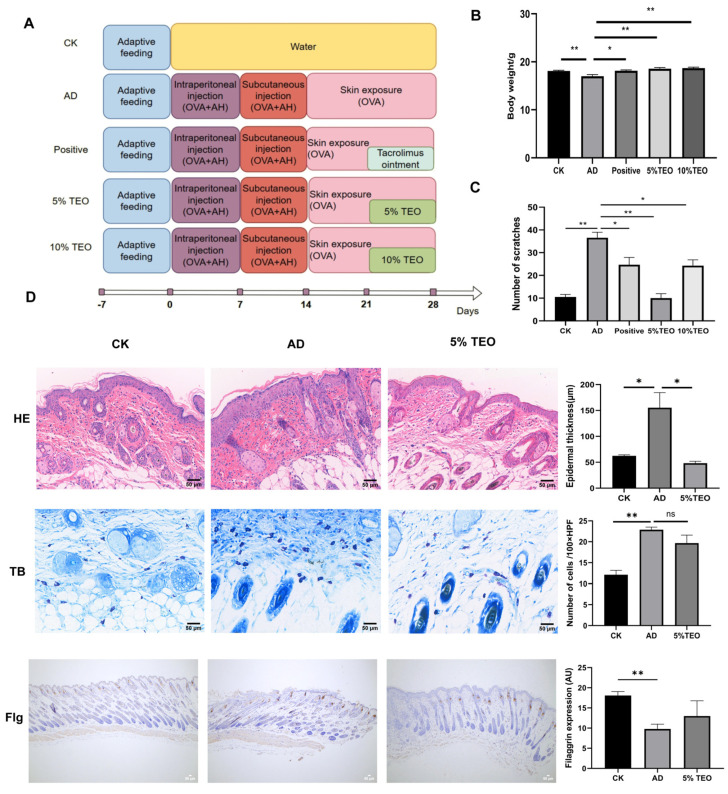
TEO prevents the development of AD in mice. (**A**) A flowchart of the animal experiment; (**B**) changes in body weight; (**C**) scratching behavior; (**D**) representative images of histological sections of the skin. Scale bar = 50 μm. Groups: CK, untreated control; AD, OVA-induced atopic dermatitis; positive, tacrolimus; 5% TEO and 10% TEO, *Thuja sutchuenensis* essential oil at 5% and 10% (*v*/*v*). Data are represented as mean ± SEM. N = 5–6 mice per group. *p* < 0.05 (*); *p* < 0.01 (**).

An AD mouse model was established using ovalbumin (OVA, MACKLIN, Shanghai, China) [18]. Specifically, after one week of acclimatization, 6–8-week-old SPF-grade C57 bl/6 mice were systemically sensitized on day 1 via intraperitoneal injection of 0.5 mL of 200 μg/mL OVA solution containing 5% alum adjuvant. On day 6, the mice were subjected to a second systemic sensitization by subcutaneous injection of 0.5 mL of 100 μg/mL OVA solution into the dorsal region. On day 17, after anesthesia with sodium pentobarbital, the dorsal hair of the mice was removed, and the first round of local sensitization was initiated. The dorsal skin was repeatedly stimulated eight times using a brush, followed by the application of 50 μL of 1000 μg/mL OVA solution onto a 0.5 cm^2^ sterile gauze pad, which was then placed on the dorsal skin. The area was covered with plastic wrap to prevent evaporation and secured with an elastic bandage. Fresh OVA solution was applied daily for one week. At this stage, the mice were randomly divided into five groups (n = 6): (1) control group (CK); (2) OVA-treated group (AD); (3) OVA plus 10% TEO group; (4) OVA plus 5% TEO group; (5) OVA plus tacrolimus (0.03% ointment, LEO Laboratories Ltd., Dublin, Ireland) group. Starting from day 39, the second round of local sensitization was performed (repeating the first-round procedure). TEO and tacrolimus ointment were administered once daily. Drug application continued for two weeks (Figure 1A). During the experiment, the mice’s food intake and mental state were observed, and body weight changes were monitored. The blank control group (CK) underwent parallel hair removal without any additional stimulation.

#### 2.3.2. Scratching Behavior Assessment

One hour after the final OVA induction, the scratching behavior of the experimental mice was observed. A valid scratch was defined as the mouse scratching the OVA-sensitized skin area. Continuous scratching for more than 3 s was counted as two scratches, and manual intervention was applied after 3 s to stop the scratching behavior. The number of scratches within a 10 min period was recorded.

#### 2.3.3. Blood Analysis

Twenty-four hours after the final administration, the mice were euthanized via intraperitoneal injection of 20% pentobarbital (Sigma-Aldrich, Burlington, MA, USA) at 3 mL/kg of body weight, before cervical dislocation. Blood samples were collected from the group of mice, and serum was separated and stored at −80 °C. Serum biochemical parameters were measured using a biochemical analyzer (Shenzhen Mindray Biomedical Electronics Co., Ltd., Shenzhen, China). The concentrations of IgE, IL-4, IL-10, IL-1β, TNF-α, and IFN-γ in the serum were determined using a biotin-labeled double-antibody sandwich enzyme-linked immunosorbent assay (ELISA) according to the manufacturer’s instructions (Wuhan Fine Biotech Co., Ltd., Wuhan, China), and the results were calculated using dedicated software.

#### 2.3.4. Skin Microbiota Composition

At the end of the experiment, microbial samples were collected from the skin wounds and surrounding areas of the mice using sterile swabs moistened with saline. For the blank control group, microbial samples were collected from the skin using sterile swabs and suspended in saline solution. DNA was extracted from the samples using a microbial DNA extraction kit (Tiangen Biotech, Beijing, China) according to the manufacturer’s instructions. The distribution of microbial species was analyzed, and the 16S rRNA gene was amplified using primers (338F: ACTCCTACGGGAGGCAGCA, 806R: GGACTACHVGGGTWTCTAAT). The sequencing and analysis of microbial composition were performed by Personal Biotechnology Co., Ltd. (Shanghai, China) on the Illumina Novaseq platform. Sequence denoising and OTU clustering were conducted using the QIIME2 pipelines. To assess the diversity level of each sample, the distribution of ASVs/OTUs across samples was evaluated, and bioinformatics analysis was performed. Differential metabolic pathways were identified based on the KEGG and COG databases using normalized pathway/group abundance tables.

#### 2.3.5. Skin Tissue Assessment

Skin tissue samples (approximately 1.0 cm × 0.5 cm) were collected from the dorsal region of the OVA-sensitized mice (from the allergen-exposed area) and the corresponding abdominal region of the non-sensitized group. The samples were fixed in 10% formaldehyde solution, embedded in paraffin, sectioned, and stained with hematoxylin-eosin (HE) (Sigma Aldrich, Burlington, MA, USA). Toluidine blue (TB) (Sigma-Aldrich, Burlington, MA, USA) staining was conducted on skin tissue to identify infiltrated mast cells. The number of TB-positive cells in four random profiled areas was used for subsequent statistical analysis. Mast cells and degranulated mast cells were counted in three randomly selected fields of view at 400× magnification for each sample. The degranulation rate was expressed as the proportion of degranulated mast cells relative to the total mast cell population. The sections were observed under Pannoramic 250 digital slice scanner (3DHISTECH, Budapest, Hungary), and images were analyzed using ImageJ 2.0 software.

#### 2.3.6. Immunohistochemistry

Tissue sections were baked in a 65 °C oven for approximately 1.5 h, cooled, and then dewaxed. The sections were placed in a container with antigen retrieval solution and heated to 95 °C for 15 min, followed by three washes with PBS (5 min each). Rabbit anti-mouse filaggrin (HUABIO, Hangzhou, China) (1:200 dilution) was added and incubated at 4 °C overnight. After three PBS washes (5 min each), goat anti-rabbit IgG (Immunoway Biotechnology Co., Ltd., Suzhou, China) working solution (1:1000 dilution) was applied and incubated at room temperature in the dark for 40 min. Streptavidin–peroxidase staining was performed according to the kit instructions. After color development with the chromogen, the sections were dried with filter paper, counterstained with hematoxylin for 30 s, differentiated with hydrochloric acid ethanol, and mounted with neutral gum. The sections were observed under a microscope, and images were analyzed using ImageJ 2.0 software.

### 2.4. Cell Culture and Cell Viability Assay

RAW 264.7 (1 × 10^6^ viable cells/mL) was resuspended in high-glucose DMEM (Solarbio, Beijing, China) containing 10% GoldORG fetal bovine serum (from Uruguay) (BBI, Shanghai, China) and 1% penicillin–streptomycin antibiotic (YEASEN, Shanghai, China). RAW 264.7 was then inoculated into the chambers of the 6-well chambers. The co-culture system was treated with TEO (20 μg/mL) and LPS (100 ng/mL; Sigma, St. Louis, MO, USA) for 24 h. Cell viability was measured by CCK-8 assay (YEASEN, Shanghai, China). RAW 264.7 cells were seeded into 96-well cell culture plates at a density of 1 × 10^4^ cells/well. After 24 h, cells were treated with 100, 50, 10, 5, 1, 0.1, 0.01, 0.001, and 0 μg/mL TEO for 24 h. Each plate contained blank wells (DMEM + CCK-8, no cells) and vehicle control wells (cells + 0.1% Tween 80). Then, CCK-8 (10 μL, 0.5 mg/mL) was added to each well and cultured for 2 h, which were measured at 450 nm using a microplate reader (Thermo, Waltham, MA, USA). Each treatment condition was measured in technical triplicate on the same plate and the experiment was independently repeated 3 times (biological replicates). After blank subtraction, viability was normalized to the vehicle control (100%). Data are reported as mean ± SEM across biological replicates.

### 2.5. Quantitative Real-Time Pcr Analysis

Total RNA was extracted from cell samples of different concentrations of RAW264.7 using the Cell/Tissue Total RNA Kit (Tiangen Biotech, Beijing, China). RNA concentration was measured with an ultra-micro spectrophotometer (Tiangen Biotech, Beijing, China), and reverse transcription was performed using PrimeScript RT Master Mix (Takara, Dalian, China).

Quantitative real-time PCR (qRT-PCR) was employed to analyze the mRNA expression of JAK1 (Forward: GACTGCAATGCCATGGCGTT, Reverse: CTTCACCTCAGTCTTCTTGA) and STAT3 (forward: TGGAGCAATACCAAAGCCGA, reverse: GCTGCAGGTCCATGTTGAAG). qRT-PCR was conducted using hieff qPCR SYBR green master mix (High Rox Plus, Yeasen, Shanghai, China) on an ABI StepOne Plus detection system (Thermo Fisher Scientific, Waltham, MA, USA). Amplification conditions were as follows: 95 °C for 5 min, followed by 40 cycles of 95 °C for 10 s, 60 °C for 20 s, and 72 °C for 20 s. Dissociation curve analysis was performed to confirm primer specificity, and β-actin (forward: TGCGTGACATTAAGGAGAA, reverse: AAGGAAGGCTGGAAGAGT) served as an internal control. The expression level of target RNA was calculated based on the threshold period (Ct), R = 2^−ΔΔCT^.

### 2.6. Western Blot Quantification

The cytoplasmic fraction containing equal amounts of protein (30 μg) were loaded and separated using 10% SDS–polyacrylamide gel electrophoresis. The proteins were then transferred onto a PVDF membrane using a trans-blot turbo transfer system (Bio-rad, Hercules, CA, USA). The membranes were washed and blocked using 5% skimmed milk. Next, the membranes were incubated overnight at 4 °C with specific primary rabbit polyclonal antibodies against JAK1 (1:1000), p-JAK1 (1:1000), STAT (1:1000), p-STAT (1:1000), and COX-2 (1:1000). The next day, the membranes were washed and then incubated with horseradish peroxidase-conjugated secondary anti-rabbit antibody. The protein recognized by the antibody was visualized using an enhanced chemiluminescence pico kit (Epizyme Biotech, Shanghai, China). The blots were stripped and re-probed for β-actin (1:5000; monoclonal antibody, mAb, HUABIO, Hangzhou, China) as a loading control. The intensity of the bands was measured using densitometry and quantified using Image J software (NIH, Bethesda, Rockville, MD, USA).

### 2.7. Network Pharmacology Analysis

The active components of TEO were screened using the ADMETlab 3.0 database and analysis platform (https://admetlab3.scbdd.com/) [19], with Lipinski’s Rule of Five (Ro5) and Veber’s criteria were applied to exclude compounds with poor oral bioavailability (MW > 500, LogP > 5, HBD > 5, HBA > 10, TPSA > 140 Å^2^), and compounds with human intestinal absorption (HIA) < 80% or P-gp substrate activity were deprioritized. Compounds with AMES mutagenicity, hERG inhibition (IC50 < 10 μM), or hepatotoxicity alerts were excluded.

Using PubChem (https://pubchem.ncbi.nlm.nih.gov/) database, the SMILES codes for each component were retrieved from TEO and submitted to the CHEMBL (https://www.ebi.ac.uk/chembl/, accessed on 3 February 2025), Swiss (http://www.swissadme.ch/), TargeNet (http://targetnet.scbdd.com/calcnet/index/, accessed on 3 February 2025) database to further explore and mine any potential targets that might be overlooked. We integrated and eliminated duplicates and integrated CHEMBL numbers and STITCH code for validation objects. To standardize the target naming method, we standardized the obtained target names using the UniProt database. Finally, we constructed a comprehensive target database, including all the merged and validated targets.

Using “Atoptic dermatitis” as the keyword, the related targets of AD were searched in GeneCards database (https://www.genecards.org/), DrugBank database (https://go.drugbank.com/), GeneMap database (https://www.omim.org/). Reducing the retrieval scope to “Homo sapiens” merged the targets, checked the structural integrity of the search result and ensured their consistency. The overlap of TEO active ingredient targets and AD genes was established using the VENNY2.1.0 website (https://bioinfogp.cnb.csic.es/tools/venny/, accessed on 3 February 2025). DAVID database (https://davidbioinformatics.nih.gov/) was used to analyze the Kyoto encyclopedia of genes and genomes (KEGG) and gene ontology (GO) pathways of intersecting targets. The results with relatively higher (*p* < 0.05) were selected for visual mapping (https://www.bioinformatics.com.cn/). STRING (https://cn.string-db.org/) was used to construct a protein–protein interaction (PPI) network. Cytoscape 3.9.1 (National Human Genome Research Institute, Bethesda, MD, USA) [20] was used with the CytoNCA plug-in to screen the targets to visualize and analyze the network topology, including the minimum and maximum degrees of freedom.

### 2.8. Molecular Docking and Molecular Dynamics

To analyze the binding affinities and modes of interaction between the drug candidate and their targets, Autodock Vina 1.2.2, a silico protein–ligand docking software, was employed [21]. The molecular structures of ENMD-2076 were retrieved from PubChem Compound (https://pubchem.ncbi.nlm.nih.gov/). The 3D coordinates of TNF-α (PDB ID, 1TNF; resolution, 2.5 Å), PTPRC (PDB ID, 5FN7; resolution, 2.3 Å), CCR5 (PDB ID, 4S2S; resolution, 2.1 Å), and JAK1 (PDB ID, 6GGH; resolution, 1.7 Å) were downloaded from the PDB (http://www.rcsb.org/pdb/home/home.do, accessed on 6 February 2025). For docking analysis, all protein and molecular files were converted into PDBQT format, with all water molecules excluded, and polar hydrogen atoms were added. The grid box was centered to cover the domain of each protein and to accommodate free molecular movement. The grid box was set to 30 Å × 30 Å × 30 Å, and grid point distance was 0.05 nm. Molecular docking studies were performed by Autodock Vina 1.2.2 (http://autodock.scripps.edu/).

After docking, the compound with the highest binding energy for each target was simulated by MD simulation to check the stability of the compound in the binding pocket [22]. Then, GROMACS 2020.6 software package, gromos54a7atb.ff force field, and a single point charge (SPC216) model was used for a molecular dynamics simulation of the 100 ns. Molecular systems were prepared with the AMBER99SB-ILDN force field for proteins and GAFF for ligands (Amber20). Solvation was performed using TIP3P water molecules in a truncated octahedral box (10 nm edge length), with ion addition (Na^+^/Cl^−^) for charge neutralization. The system underwent energy minimization using 2500 steps of steepest descent followed by 2500 steps of conjugate gradient algorithms. Subsequently, equilibration was performed under the following protocol: (1) 100 ps of NVT ensemble simulation at 298.15 K and (2) 100 ps of NPT ensemble simulation, with temperature maintained at 298.15 K. Production molecular dynamics was then conducted for 100 ns under NPT ensemble conditions with periodic boundary constraints. Long-range electrostatic interactions were calculated using the Particle Mesh Ewald (PME) method with a 1.0 nm cutoff for non-bonded interactions. The simulation parameters included the following: collision frequency of 2/ps, constant pressure of 101.325 kPa (1 atm), integration time step of 2 fs, and trajectory-saving interval of 10 ps.

### 2.9. Statistical Analysis

Statistical analysis was performed using GraphPad Prism 8 software, Student’s *t*-test was employed to compare two groups, and one-way ANOVA was used to compare multiple groups. The experimental data came from at least three repetitions and were expressed as the mean ± SEM. *p* < 0.05 was considered statistically significant (* *p* < 0.05; ** *p* < 0.01; *** *p* < 0.001).

## 3. Results

### 3.1. Teo Reduces Ova-Induced AD-like Changes

As expected, repeated applications of OVA to the skin induced AD-like skin lesions, including erythema, desquamation, edema and scratch marks in mice (Figure 1A). Throughout the experimental period, the body weight of AD mice was significantly lower than that of the other groups (Figure 1B). We observed that OVA notably stimulated hyperplasia, hyperkeratosis, and parakeratosis in the epidermis and dermis and also enhanced the scratching behavior of the mice. The severity of lesions was significantly decreased following TEO (10%, 5%) and tacrolimus ointment of the emollient compared to the AD mice. The behavioral assessment revealed a marked decrease in scratching frequency, particularly in mice treated with 5% TEO, which showed the least scratching activity. The application of 5% TEO largely restored the histological alterations in the skin, evidenced by reductions in epidermal thickening and inflammatory cell infiltration (Figure 1C). While a decrease in mast cell numbers was noted, it did not reach statistical significance compared to AD mice, and TEO had no significant influence on mast cell degranulation (Figure 1D). Filaggrin is found in the skin, protecting against external stimuli; filaggrin expression appears abnormal in damaged skin tissue. We detected the expression of filaggrin via immunohistochemical staining. It can be seen that TEO can improve the abnormal proliferation and differentiation of keratin in filaggrin (Figure 1D).

### 3.2. Teo Regulates Serum Biochemical Parameters in AD Mice

**Figure 2:**
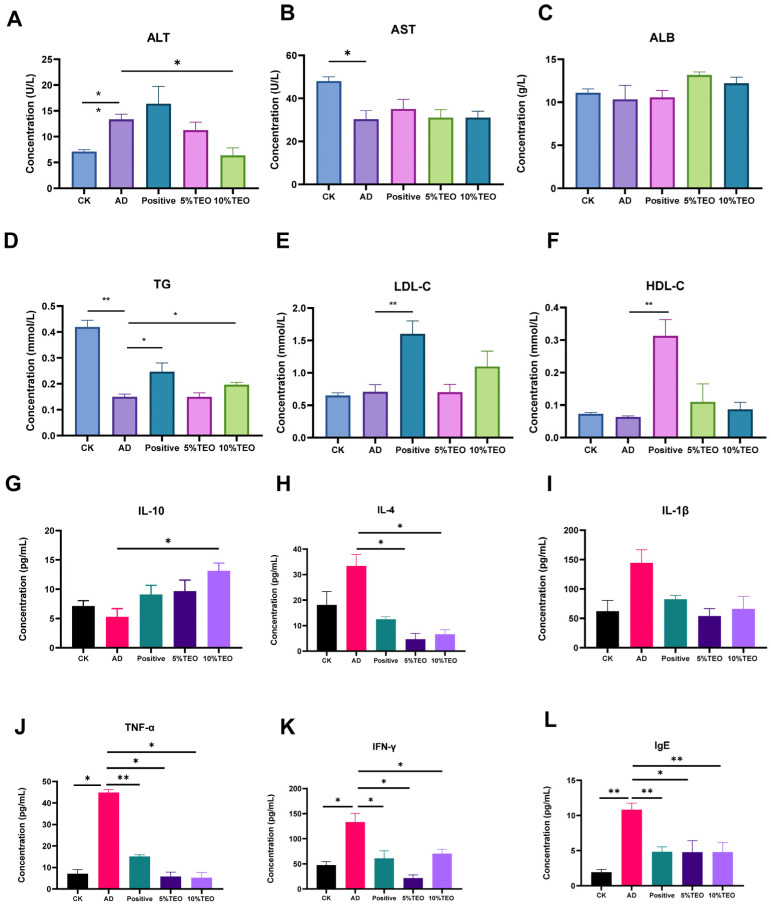
Modulatory effects of TEO on hematological profiles in mice. (**A**) ALT (U/L), (**B**) AST (U/L), (**C**) ALB (g/L), (**D**) TG (mmol/L), (**E**) LDL-C (mmol/L), (**F**) HDL-C (mmol/L), (**G**) IL-10, (**H**) IL-4, (**I**) IL-1β, (**J**) TNF-α, (**K**) IFN-γ, (**L**) IgE (pg/mL). Groups: CK, untreated control; AD, OVA-induced atopic dermatitis; Positive, tacrolimus; 5% TEO and 10% TEO, *Thuja sutchuenensis* essential oil at 5% and 10% (*v*/*v*). Bars represent mean ± SEM (n = 5–6). Statistical significance: *p* < 0.05 (*), *p* < 0.01 (**).

A biochemical parameters analysis of mice with AD showed that, compared with the CK group, the AD group maintained higher levels of ALT (7.113 ± 0.372), but lower levels of LDL-C (0.71 ± 0.108), AST (30.33 ± 3.844), ALB (10.33 ± 1.634), TG (0.15 ± 0.025), and HDL-C (0.063 ± 0.003). These changes were corrected upon TEO intervention, as evidenced by ALT (10%, 6.367 ± 1.484; 5%, 11.28 ± 1.538) and TG (10%, 0.197 ± 0.009; 5%, 0.15 ± 0.015) levels reaching a significant difference from the AD group. AST and ALB showed partial restoration, and LDL-C and HDL-C levels were higher than those in other groups, notably exceeding those found in the positive control group (Figure 2).

### 3.3. Teo Suppresses the Cytokine Response in AD Mice

To determine whether TEO regulates the inflammatory response in AD-like allergic dermatitis, we measured serum cytokines and chemokines in OVA-induced AD mice. Compared with the control group, topical OVA markedly increased IgE (AD: 10.82 ± 0.0935 vs. CK: 1.937 ± 0.382), IL-4 (33.37 ± 4.417 vs. 18.15 ± 5.248); IL-1β (144.5 ± 22.27 vs. 62.31 ± 18.32); IL-10 (5.267 ± 1.424 vs. 7.13 ± 0.934); TNF-α (44.88 ± 1.486 vs. 7.114 ± 1.898); and IFN-γ (133.2 ± 17.26 vs. 47.64 ± 7.025). Topical TEO 5% and TEO 10% reduced IgE to 4.768 ± 1.650, 4.776 ± 1.353; IL-4 to 4.693 ± 2.299, 6.629 ± 1.784; TNF-α to 5.865 ± 2.009, 5.283 ± 2.375; and IFN-γ to 21.72 ± 6.392, 70.26 ± 8.654, while IL-1β remained at 5%: 53.98 ± 12.64; 10%: 66.20 ± 21.41. Consistent with an anti-inflammatory effect, IL-10 increased with TEO to 5%: 9.698 ± 1.852; 10%: 13.12 ± 1.316, AD: 5.267 ± 1.424. Interestingly, tacrolimus treatment did not reduce the levels of TNF-α, although it did reduce the other levels (Figure 2).

### 3.4. Teo Restores Skin Microbiota Homeostasis in AD Mice

**Figure 3:**
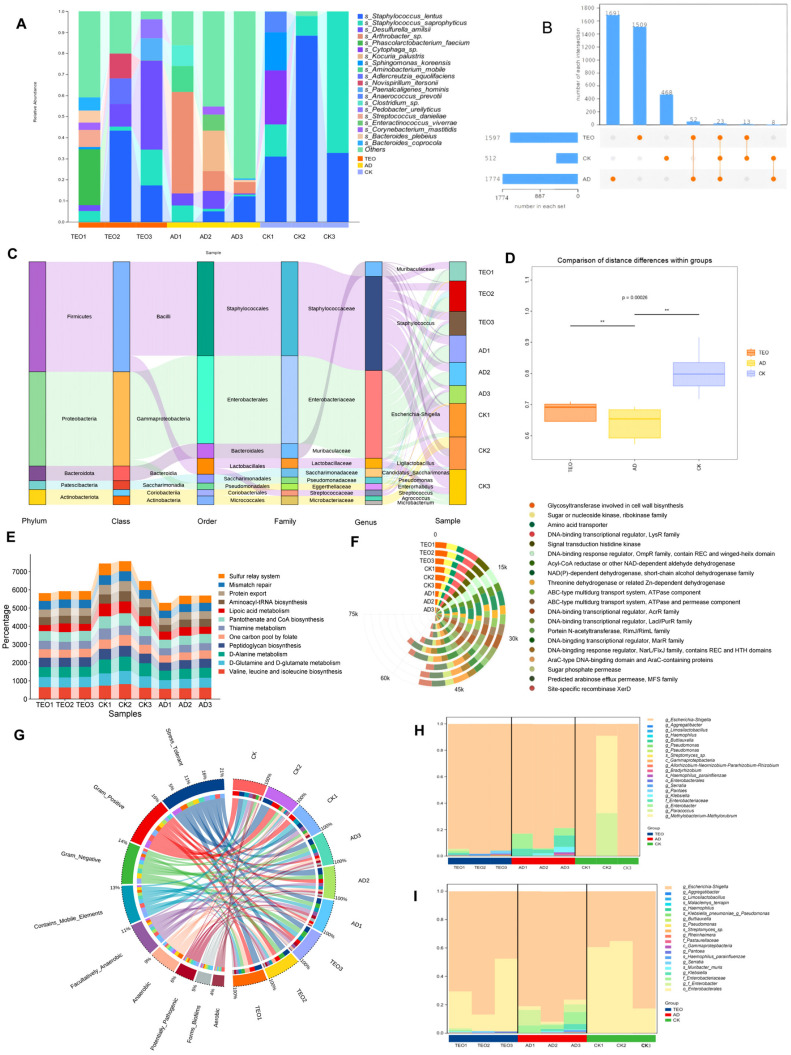
Effects of TEO treatment on skin microbiota composition and functional analysis in AD mice: (**A**) relative abundance of dominant microbial genera; (**B**) Venn diagram showing shared and unique microbial species; (**C**) Sankey diagram illustrating the taxonomic composition and relative abundance of skin microbiota; (**D**) diversity analysis of skin microbiota; (**E**) KEGG pathway enrichment analysis; (**F**) GO functional annotation; (**G**) phenotypic characterization of skin microbiota using BugBase analysis; (**H**,**I**) species contribution to the forms’ biofilms and potentially pathogenic phenotype. Groups: CK, untreated control; AD, OVA-induced atopic dermatitis; TEO, *Thuja sutchuenensis* essential oil at 5% (*v*/*v*) (n = 5–6).

*Staphylococcus* and *Escherichia-Shigella* were identified as the predominant genera based on high-throughput sequencing analysis (Figure 3C). The skin microbiota of the CK group showed limited diversity and was primarily composed of *Staphylococcus lentus* and *Staphylococcus saprophyticus*. In comparison, AD- and TEO-treated mice exhibited greater microbial diversity, but this was accompanied by a rise in harmful microorganisms, including *Arthrobacter* sp., which was uniquely associated with the AD group. The Venn diagram analysis identified 23 microbial species common to all groups, with the AD group containing the most unique species and the CK group containing the fewest. In addition, significant differences in microbial composition were observed among the groups (Figure 3A,B,D). An elevated abundance of harmful and conditionally pathogenic microbes, including *Corynebacterium mastitidis*, *Streptococcus danieliae*, *Leptotrichia wadei*, *Desulfurella amilsii*, and *Cytophaga* sp., was detected in the AD and TEO groups (Figure 3A).

Using machine learning, the roles of these microorganisms in the KEGG and GO pathways were analyzed, and the top 20 most significant processes were graphically represented. The KEGG pathways were categorized into metabolism and genetic information processing. The results indicated that the CK group had the highest abundance in several pathways, including D-Glutamine and D-glutamate metabolism, D-Alanine metabolism, lipoic acid metabolism, pantothenate and CoA biosynthesis, and the sulfur relay system, followed by the TEO group (Figure 3E). According to GO analysis, cellular processes and signaling, metabolism, and information storage and processing were the predominant enriched biological processes, in agreement with previous analyses, and the CK group displayed the highest activity among all groups (Figure 3F). NAD(P)-dependent dehydrogenase, short-chain alcohol dehydrogenase family, predicted arabinose efflux permease (MFS family), DNA-binding transcriptional regulator (MarR family), and ABC-type multidrug transport system (ATPase component) were more abundant in the CK group, with the TEO group showing intermediate levels compared to the CK and AD groups. Furthermore, a phenotypic characterization of the skin microbiota was conducted using the BugBase database (Figure 3G). The results highlighted that Gram-positive bacteria displayed the largest disparity, with the CK group showing the highest abundance and the AD group the lowest. Forms’ biofilms and potentially pathogenic phenotypes were also most abundant in the AD group, with the TEO group exhibiting intermediate levels. Furthermore, biofilms associated with disease susceptibility and potential pathogenic microorganisms were primarily colonized by taxa from the TEO and AD groups, with a significantly lower abundance observed in the CK group (Figure 3H,I).

### 3.5. Network Pharmacology Prediction of Teo for AD

**Figure 4:**
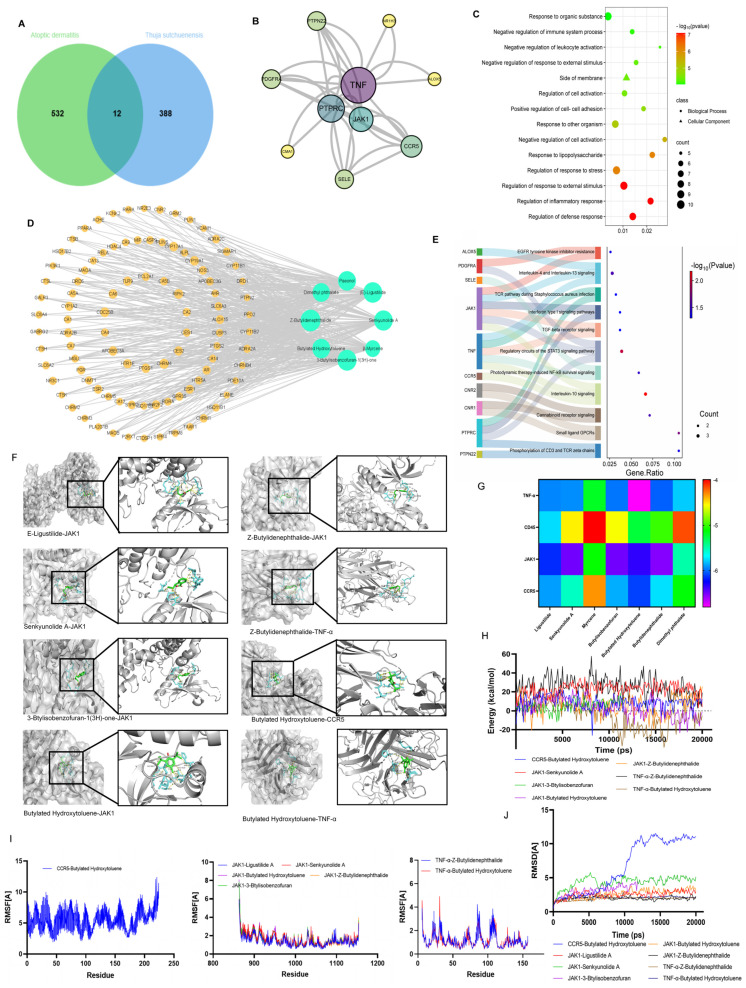
Network pharmacology prediction of TEO for AD. (**A**) Venn diagram of common target genes between TEO and AD; (**B**) the result of a PPI analysis for potential targets of TEO; (**C**) GO analysis selecting three parts, including the biological process and cellular composition; (**D**) network diagram of “TEO–ingredient–target–AD” interaction; (**E**) the result of KEGG enrichment analysis for the potential pathway of TEO; (**F**) molecular docking of active compounds with target proteins; (**G**) binding energy of active product–target docking complexes; (**H**) binding energy of the complex; (**I**) residue-wise root–mean–square fluctuation (RMSF) of the molecular complex; (**J**) atomic-level root–mean–square deviation (RMSD) of the molecular complex.

GC-MS analysis revealed that TEO comprises 110 principal constituents (Table A1). To explore the therapeutic mechanism of TEO in AD, network pharmacology was employed to analyze its potential targets. The results showed that 24 active components (Table 1), 408 drug targets, and 546 disease targets were identified. The active ingredients and AD targets were entered into the VENNY2.1.0 website, and 12 intersecting targets were identified (Figure 4A). A chemical–target interaction network diagram was drawn to visually analyze the active components in the drug and the drug–disease target (Figure 4D). The PPI network was visualized using Cytoscape, and according to the degree, TNF, PTPRC, CCR5, JAK1, and other genes were identified as core genes (Figure 4B).

GO enrichment analysis of the 12 overlapping genes included regulation of inflammatory response, negative regulation of response to external stimulus, positive regulation of cell–cell adhesion, regulation of response to stress, and response to lipopolysaccharide (Figure 4C). The KEGG pathways associated with AD were mainly involved in the IL-10 signaling pathway, IL-4 and IL-13 signaling, TGF-beta receptor signaling, the TCR pathway during *Staphylococcus aureus* infection signaling, and the STAT3 pathways (Figure 4E).

To assess the binding affinity of the candidate compounds for their targets, we performed a molecular docking analysis. The binding poses and interactions of eight compounds with four proteins were obtained with Autodock Vina v.1.2.2 and the binding energy for each interaction was generated (Figure 4G). The results showed that each drug candidate bound to its protein target through visible hydrogen bonds and strong electrostatic interactions. Moreover, the hydrophobic pockets of each target were successfully occupied by the eight candidate drugs. For JAK1, five candidates ((E)-ligustilide, senkyunolide A, 3-butylisobenzofuran-1(3H)-one, butylated hydroxytoluene, and Z-buthlidenephthalide) had low binding energy, indicating highly stable binding.

To elucidate the dynamic interactions between receptor proteins and small molecules during exercise and evaluate binding site stability, we performed 100 ps molecular dynamics (MD) simulations, with subsequent validation of system equilibration. The root–mean–square deviation (RMSD) analysis of the complex system is presented in Figure 4J. Both complex systems (JAK1-ligustilidebut and JAK1-hlidenephthalide) rapidly achieved equilibrium in MD simulations, exhibiting comparable energy fluctuation patterns, which collectively indicates stable molecular binding (Figure 4H). Root–mean–square fluctuation (RMSF) analysis quantitatively evaluates residue-specific flexibility by considering the positional deviations of individual amino acids throughout the simulation trajectory. As shown in Figure 4I, RMSF profiling of both systems revealed nearly identical variation patterns across all residues. Notably, all measured RMSF values remained below 2 Å, demonstrating the exceptional structural rigidity and dynamic stability of the protein backbone. These findings strongly suggest that the protein maintains remarkable conformational stability during functional dynamics, exhibiting both the structural resilience and environmental adaptability essential for proper biological function.

### 3.6. Teo Attenuates Lps-Induced Inflammation in Raw 264.7 by Suppressing JAK1/STAT3 Signaling

**Figure 5:**
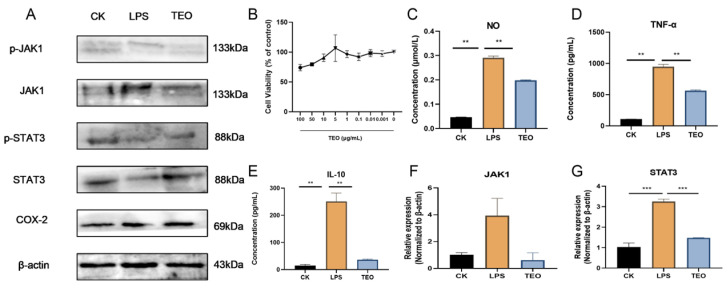
TEO exhibited potent anti-inflammatory effects in vitro, attenuating JAK1/STAT3 signaling pathways in LPS cell models. (**A**) Western blot analysis of JAK1/STAT3 pathway activation and COX-2 expression; (**B**) TEO modulates RAW264.7 cellular activity; (**C**–**E**) TEO significantly reduced LPS-induced production of NO, TNF-α, and IL-10 in RAW264.7; (**F**,**G**) relative mRNA expression of JAK1 and STAT3. Groups: CK, untreated control; LPS, 100 ng/mL LPS-induced; TEO, 100 ng/mL LPS-induced and *Thuja sutchuenensis* essential oil at 20 μg/mL. Bars represent mean ± SEM (n = 3). Statistical significance: *p* < 0.01 (**), *p*< 0.001 (***).

The CCK8 assay showed that TEO had no significant cytotoxic effects on RAW 264.7 cells at concentrations of 0–100 μg/mL (Figure 5B). After stimulation by LPS, the NO, TNF-α, and IL-10 production increased in RAW264.7 cells, but treatment with 20 μg/mL significantly inhibited the protein levels of NO, TNF-α, IL-1β, and IL-10 (Figure 5C–E). The protein levels of JAK1, p-JAK1, STAT3, p-STAT3, and COX-2 were significantly reduced by TEO treatment in LPS-stimulated RAW 264.7 cells (Figure 5A); JAK1 and STAT3 mRNA expression was also reduced, which further confirmed the inhibitory effect of TEO on the JAK1/STAT3 pathway.

## 4. Discussion

Atopic dermatitis represents a multifactorial systemic inflammatory disease with distinct cutaneous manifestations, classically presenting with a clinical triad of xerosis, chronic eczematous eruptions, and intractable pruritus. The histopathological hallmarks comprise a mixed dermal inflammatory infiltrate (predominantly macrophages, neutrophils, and polarized Th2/Th1 lymphocytes) accompanied by marked epidermal hyperplasia with aberrant differentiation [23]. Our findings demonstrate that topical application of TEO significantly attenuates AD-like allergic skin inflammation and reduces epidermal hyperplasia in experimental models. While the exact etiology of atopic dermatitis is still being elucidated, emerging evidence highlights the interplay between immunological abnormalities, impaired epidermal barrier function, and microbial dysbiosis as central to disease pathogenesis [2]. Our approach, combining microbiota sequencing, functional annotation, and phenotypic characterization, uncovered marked dysbiosis in AD-associated skin microbiota and suggested TEO’s therapeutic efficacy. The AD group showed selective enrichment of pathogenic taxa (e.g., *Arthrobacter* sp. and *Corynebacterium mastitidis*) and upregulated biofilm formation. Mechanistically, this dysbiosis may promote inflammation via TLR2/4 activation by *Staphylococcus aureus*, triggering Th2 cytokine (IL-4/IL-13) release [24]. Our integrated experimental results corroborate this theoretical framework, with network pharmacology analysis further delineating critical nodal points (TLR2-IL4R-STAT3 axis) within the disease cascade. Concurrently, biofilm-mediated protection and dysregulated D-amino acid/lipoic acid metabolism pathways likely impair barrier function and perpetuate allergen infiltration [25]. TEO treatment partially restored skin microbiota diversity in AD mice, but the elevated abundance of certain opportunistic pathogens (e.g., *Desulfurella amilsii*) at higher levels than the CK group suggest that the treated mice may still be undergoing recovery. The terpenoid compounds in TEO (e.g., terpinen-4-ol) may reduce biofilm formation by disrupting bacterial cell membranes and inhibiting quorum sensing, as demonstrated by the antibacterial and antibiofilm activities of terpenoids against pathogens [26,27]. Additionally, the restoration of lipid metabolism (e.g., lipoic acid metabolism) and energy metabolism pathways in the TEO group may enhance skin barrier function and reduce oxidative stress [28]. Among the major constituents of TEO (β-caryophyllene, caryophylleneoxide, linalool, α-terpineol, and paeonol), several have mechanistic links to AD-relevant pathways. β-Caryophyllene (BCP)—a selective CB_2_ agonist—ameliorates DNCB-induced AD-like dermatitis and downregulates keratinocyte TSLP/EGR1, supporting antipruritic and anti-inflammatory actions consistent with our cytokine trends [29]. In addition, topical BCP is reported to suppress NF-κB/MAPK signaling and bolster antioxidant defenses in skin, further aligning with AD pathophysiology [30]. Major monoterpene alcohols, such as linalool and α-terpineol, can attenuate macrophage/keratinocyte inflammatory outputs via the NF-κB/MAPK interference, which provides a plausible basis for the reductions we observe in Th2-skewed mediators [31]. Finally, the phenolic paeonol improves DNCB-induced AD-like lesions in mice by lowering IgE and Th2 cytokines and modulating mast and T-cell responses, supporting a complementary anti-inflammatory contribution [32]. Furthermore, TEO likely alleviates inflammation by suppressing the TLR2/STAT3 signaling pathway, as evidenced by the anti-inflammatory effects of Cupressaceae terpenoids in atopic dermatitis models [33]. However, the regulatory effects of TEO on Th2 cytokines require further validation.

In the complex pathophysiology of atopic dermatitis, TNF-α emerges as a pivotal pro-inflammatory cytokine that compromises skin barrier integrity while potentiating Th2-type immunity [24]. This inflammatory milieu is further amplified through the CCR5-mediated recruitment of Th1/Th17 cells [34] and CD45-dependent leukocyte activation. Critically, the JAK1-STAT3 axis serves as a signaling nexus, where JAK1 phosphorylates STAT3 to transduce Th2 cytokine signals (IL-4/IL-13) [1] while simultaneously orchestrating inflammatory responses and barrier repair [35]. Previous studies have shown that the expression of phosphorylated STAT3 and p65 is significantly upregulated in the AD group, which is consistent with the enhanced inflammation and increased immune cell infiltration [34]. COX-2, the pivotal inflammatory enzyme responsible for prostaglandin (e.g., PGE2) production, is regulated by JAK1-STAT3 signaling. STAT3 inhibition markedly suppresses COX-2 expression in LPS-stimulated macrophages [36]. Notably, TEO treatment similarly reduced COX-2 levels, paralleling STAT3 activity changes. TEO treatment markedly reduced the expression of these proteins, suggesting its anti-inflammatory effects through inhibition of the JAK-STAT signaling pathways. Specifically, the downregulation of phosphorylated STAT3 indicates that TEO may alleviate Th2-type inflammation. These findings align with the observed restoration of skin barrier function and the reduction in oxidative stress in TEO-treated mice.

Inflammatory responses do not function through independent pathways, but are typically maintained by the collaboration of multiple pathways. Upon activation of the JAK-STAT pathway, negative feedback proteins (such as SOCS1/3) are induced, which bind directly to JAK or STAT to inhibit their activity, while also suppressing the transcription of JAK/STAT genes [37]. Although STAT mRNA is reduced, existing STAT proteins may be phosphorylated by other kinases (such as Src family kinases) due to persistent inflammatory stimuli (e.g., IL-6, IFN-γ), resulting in an elevated level of p-STAT. The activation (phosphorylation) of STAT is rapid and transient, and its function primarily depends on post-translational modifications rather than the synthesis of new proteins. Even with reduced STAT mRNA levels, cytokines in the inflammatory microenvironment can maintain signaling through the phosphorylation of residual STAT proteins [38]. Cross-talk regulation (e.g., the activation of STAT3 through non-JAK-dependent pathways such as MAPK and EGFR) leads to the phosphorylation of STAT3 in certain inflammatory models (e.g., TNF-α stimulation), where STAT3 phosphorylation can occur via non-JAK-dependent pathways (such as MAPK and EGFR pathways), resulting in the upregulation of p-STAT, even in the absence or downregulation of JAK activation [39]. In addition, IL-10 exerts its anti-inflammatory effects by activating STAT3; however, its action may suppress the expression of pro-inflammatory JAKs, such as JAK1, leading to a decrease in JAK mRNA levels while concomitantly increasing the phosphorylation of STAT3 [40]. This study identifies an inhibitory effect of TEO on AD, but several limitations remain—namely, reliance on a single murine model, a short exposure period, and the lack of pharmacokinetic and pediatric clinical data. Future work should include dose–response studies and long-term safety and efficacy trials—particularly in pediatric populations—to more systematically delineate TEO’s therapeutic efficacy and mechanisms of action.

## 5. Conclusions

It is well-established that atopic dermatitis represents a chronic, relapsing systemic atopic disorder with complex pathophysiology. Our study demonstrates that TEO alleviates key hallmarks of ovalbumin-induced AD-like dermatitis in mice—including chronic pruritus, epidermal hyperplasia, cutaneous dysbiosis, and inflammatory infiltration—through the modulation of IL-4-mediated JAK/STAT signaling. Complementary network pharmacology and in vitro validation in LPS-stimulated RAW264.7 macrophages corroborate TEO’s anti-inflammatory efficacy via JAK/STAT pathway inhibition. These integrated findings identify TEO as a promising multi-target therapeutic candidate for clinical AD management.

## Figures and Tables

**Table 1:** The active ingredient of essential oil of *Thuja sutchuenensis*.

NO	Library	MF	Structural Class	CAS	Peak Area (%)	SMILES
1	γ-Terpinene	C_10_H_16_	Monocyclic monoterpene	99-85-4	33.3779	CC1=CCC(=CC1)C(C)C
2	β-Myrcene	C_10_H_16_	Acyclic monoterpene	123-35-3	7.0674	CC(=CCCC(=C)C=C)C
3	(E)-Ligustilide	C_12_H_14_O_2_	Phthalide	81944-08-3	5.7084	CCC/C=C\1/C2=C(C=CCC2)C(=O)O1
4	3-Carene	C_10_H_16_	Bicyclic monoterpene	13466-78-9	3.1011	CC1=CCC2C(C1)C2(C)C
5	Linalool	C_10_H_18_O	Oxygenated monoterpene	78-70-6	3.0748	CC(=CCCC(C)(C=C)O)C
6	Fenchyl acetate	C_12_H_20_O_2_	Oxygenated monoterpene	13851-11-1	2.9793	CC(=O)OC1C(C2CCC1(C2)C)(C)C
7	Bicyclo [2.2.1]heptan-2-ol, 1,7,7-trimethyl-, acetate, (1S-endo)	C_12_H_20_O_2_	Oxygenated monoterpene	5655-61-8	2.4582	CC(=O)O[C@@H]1C[C@@H]2CC[C@]1(C2(C)C)C
8	D-Limonene	C_10_H_16_	Monocyclic monoterpene	5989-27-5	2.4024	CC1=CC[C@@H](CC1)C(=C)C
9	Caryophyllene	C_15_H_24_	Bicyclic sesquiterpene	87-44-5	2.0254	C/C/1=C\CCC(=C)[C@H]2CC([C@@H]2CC1)(C)C
10	β-pinene	C_10_H_16_	Bicyclic monoterpene	18172-67-3	1.7858	CC1(C2CCC(=C)C1C2)C
11	1,4,7,-Cycloundecatriene, 1,5,9,9-tetramethyl-, Z,Z,Z-	C_15_H_24_	Monocyclic sesquiterpene	1000062-61-9	1.307	CC1=CCC=C(C)CC=CC(C)(C)CC1
12	Z-Butylidenephthalide	C_12_H_12_O_2_	Phthalide	72917-31-8	1.231	CCC/C=C\1/C2=CC=CC=C2C(=O)O1
13	Longiborneol	C_15_H_26_O	Oxygenated sesquiterpene	465-24-7	0.9812	CC1(CCCC2(C3C1[C@@H](C2(CC3)C)O)C)C
14	Paeonol	C_9_H_10_O_3_	Phenolic	552-41-0	0.9512	CC(=O)C1=C(C=C(C=C1)OC)O
15	β-Bisabolene	C_15_H_24_	Acyclic sesquiterpene	495-61-4	0.903	CC1=CC[C@H](CC1)C(=C)CCC=C(C)C
16	β-Sesquiphellandrene	C_15_H_24_	Acyclic sesquiterpene	20307-83-9	0.8934	C[C@@H](CCC=C(C)C)[C@H]1CCC(=C)C=C1
17	Senkyunolide A	C_12_H_16_O_2_	Phthalide	63038-10-8	0.8522	CCCC[C@H]1C2=C(C=CCC2)C(=O)O1
18	3-Butylisobenzofuran-1(3H)-one	C_12_H_14_O_2_	Phthalide	6066-49-5	0.8441	CCCCC1C2=CC=CC=C2C(=O)O1
19	α-Curcumene	C_15_H_22_	Acyclic sesquiterpene	644-30-4	0.8387	CC1=CC=C(C=C1)C(C)CCC=C(C)C
20	(-)-α-Cedrene	C_15_H_24_	Bicyclic sesquiterpene	469-61-4	0.7608	C[C@@H]1CC[C@@H]2[C@]13CC=C([C@H](C3)C2(C)C)C
21	Longifolene	C_15_H_24_	Bicyclic sesquiterpene	475-20-7	0.7458	C[C@]12CCCC([C@H]3[C@H]1CC[C@@H]3C2=C)(C)C
22	Caryophyllene oxide	C_15_H_24_O	Oxygenated sesquiterpene	1139-30-6	0.6608	C[C@@]12CC[C@@H]3[C@H](CC3(C)C)C(=C)CC[C@H]1O2
23	(+)-α-terpineol	C_10_H_18_O	Oxygenated monoterpene	7785-53-7	0.6039	CC1=CC[C@@H](CC1)C(C)(C)O
24	γ-Himachalene	C_15_H_24_	Bicyclic sesquiterpene	53111-25-4	0.5088	CC1=C[C@H]2[C@@H](CC1)C(=CCCC2(C)C)C

## Data Availability

The original contributions presented in this study are included in the article. Further inquiries can be directed to the corresponding author.

## Abbreviations

AD	Atopic dermatitis
ALT	Alanine aminotransferase
AST	Aspartate aminotransferase
ALB	Albumin
CCR-5	C-C chemokine receptor type 5
COX-2	Cyclooxygenase 2
DMEM	Dulbecco’s modified eagle medium
EGFR	Epidermal growth factor receptor
Flg	Filaggrin
HE	Hematoxylin–eosin
HDL-C	High density lipoprotein cholesterol
IL-13	Interleukin-13
IL-4	Interleukin-4
IL-10	Interleukin-10
IL-1β	Interleukin-1β
IgE	Immunoglobulin E
IgG	Immunoglobulin G
IFN-γ	Interferon-γ
JAK	Janus kinase
LDL-C	Low-density lipoprotein cholesterol
MAPK	Mitogen-activated protein kinase
MD	Molecular dynamics
NF-κB	Nuclear factor kappa-B
OVA	Ovalbumin
PTPRC	Receptor-type tyrosine-protein phosphatase C
PPI	Protein–protein interaction
RMSD	Root–mean–square deviation
RMSF	Root–mean–square fluctuation
SOCS1/3	Suppressor of cytokine signaling 1/3
STAT	Signal transducer and activator of transcription
TB	Toluidine blue
TCR	T-cell receptor
TEO	*Thuja sutchuenensis* Franch. Essential Oil
TNF-α	Tumor necrosis factor-α
TG	Triglyceride
KEGG	Kyoto encyclopedia of genes and genomes
GO	Gene ontology

## Appendix A

**Table A1:** Analysis of essential oil of *Thuja sutchuenensis*.

NO	RT	Library	MF	Ref	CAS	Qual
1	3.813	p-Xylene	C_8_H_10_	5631	106-42-3	95
2	5.3589	γ-Terpinene	C_10_H_16_	17,501	99-85-4	90
3	5.7149	3-Carene	C_10_H_16_	17,457	13466-78-9	91
4	6.7463	β-pinene	C_10_H_16_	17,719	18172-67-3	97
5	7.3266	β-Myrcene	C_10_H_16_	17,490	123-35-3	96
6	9.2372	D-Limonene	C_10_H_16_	17,468	5989-27-5	99
7	9.805	trans-β-Ocimene	C_10_H_16_	17,523	3779-61-1	93
8	10.4796	β-Ocimene	C_10_H_16_	17,485	13877-91-3	95
9	12.9332	2-Nonanone	C_9_H_18_O	22,228	821-55-6	91
10	13.4666	Linalool	C_10_H_18_O	29,730	78-70-6	97
11	14.3915	(E)-para-2-menthen-1-ol	C_10_H_18_O	30,100	29803-81-4	93
12	14.7275	1,4-Hexadiene, 5-methyl-3-(1-methylethylidene)	C_10_H_16_	17,671	113687-24-4	95
13	15.2602	(+)-2-Bornanone	C_10_H_16_O	28,038	464-49-3	97
14	15.8664	n-Amylbenzene	C_11_H_16_	25,418	538-68-1	93
15	16.3263	endo-Borneol	C_10_H_18_O	29,771	507-70-0	97
16	16.4436	α-phellandren-8-ol	C_10_H_16_O	28,061	1686-20-0	86
17	16.7744	Terpinen-4-ol	C_10_H_18_O	29,794	562-74-3	96
18	17.3717	(+)-α-terpineol	C_10_H_18_O	30,147	7785-53-7	90
19	17.9409	(-)-Verbenone	C_10_H_14_O	26,717	1196-01-6	98
20	18.3181	Fenchyl acetate	C_12_H_20_O_2_	66,894	13851-11-1	99
21	18.7694	Citronellol	C_10_H_20_O	31,575	106-22-9	98
22	18.8572	Bicyclo [3.1.1]hept-2-en-6-ol, 2,7,7-trimethyl-, acetate, [1S-(1.alpha.,5.alpha.,6.beta.)]-	C_12_H_18_O_2_	64,905	50764-55-1	87
23	19.6085	Linalyl acetate	C_12_H_20_O_2_	66,887	115-95-7	91
24	19.6606	Geraniol	C_10_H_18_O	29,729	106-24-1	96
25	19.7834	Methyl citronellate	C_11_H_20_O_2_	55,451	2270-60-2	95
26	20.1424	Geranial	C_10_H_16_O	28,159	141-27-5	91
27	20.3388	(Z)-Undec-6-en-2-one	C_11_H_20_O	41,321	107853-70-3	95
28	20.5797	Bicyclo[2.2.1]heptan-2-ol, 1,7,7-trimethyl-, acetate, (1S-endo)-	C_10_H_18_O	67,036	5655-61-8	99
29	20.8713	2-Undecanone	C_11_H_22_O	43,210	112-12-9	96
30	21.0343	trans-Pinocarvyl acetate	C_12_H_18_O_2_	64,731	1686-15-3	83
31	21.6221	Phthalic anhydride	C_8_H_4_O_3_	25,847	85-44-9	95
32	21.808	Decanoic acid, methyl ester	C_11_H_22_O_2_	57,296	110-42-9	86
33	22.3384	1H-Indene-4-carboxaldehyde, 2,3-dihydro-	C_10_H_10_O	24,097	51932-70-8	91
34	22.5552	1,3-Cyclohexadiene, 1-methyl-4-(1-methylethyl)-	C_10_H_16_	17,678	99-86-5	94
35	22.6838	2,4,6-Cycloheptatrien-1-one	C_7_H_6_O	5627	539-80-0	93
36	22.9905	β-Bisabolene	C_15_H_24_	74,662	495-61-4	93
37	23.3117	Copaene	C_15_H_24_	74,561	3856-25-5	98
38	23.4286	α-Amorphene	C_15_H_24_	74,835	483-75-0	89
39	23.5437	Geranyl acetate	C_12_H_20_O_2_	66,885	105-87-3	91
40	23.7192	γ-Muurolene	C_15_H_24_	74,670	30021-74-0	99
41	23.8015	β-Elemene	C_15_H_24_	74,940	515-13-9	91
42	23.9956	Aromandendrene	C_15_H_24_	74,618	489-39-4	98
43	24.0693	Di-epi-α-cedrene	C_15_H_24_	74,706	50894-66-1	93
44	24.1662	Longifolene	C_15_H_24_	74,595	475-20-7	99
45	24.4161	(1R,5R)-1-Isopropyl-8-methyl-4-methylenespiro[4.5]dec-7-ene	C_15_H_24_	74,791	55732-78-0	86
46	24.5724	Caryophyllene	C_15_H_24_	74,603	87-44-5	99
47	24.7056	3,5,5,9-Tetramethyl-4a,5,6,7,8,9-hexahydro-2H-benzo[7]annulene	C_15_H_24_	74,804	1000412-94-8	87
48	24.8758	cis-Thujopsene	C_15_H_24_	74,623	470-40-6	99
49	25.2532	Paeonol	C_9_H_10_O_3_	40,302	552-41-0	95
50	25.3652	α-himachalene	C_15_H_24_	74,937	3853-83-6	99
51	25.5049	1,4,7,-Cycloundecatriene, 1,5,9,9-tetramethyl-, Z,Z,Z-	C_15_H_24_	74,761	1000062-61-9	98
52	25.5731	Dimethyl phthalate	C_10_H_10_O_4_	64,177	131-11-3	95
53	25.839	(1R,4R,5S)-1,8-Dimethyl-4-(prop-1-en-2-yl)spiro[4.5]dec-7-ene	C_15_H_24_	74,799	729602-94-2	98
54	26.0591	Amorphadiene	C_15_H_24_	74,926	92692-39-2	93
55	26.1456	γ-Himachalene	C_15_H_24_	74,879	53111-25-4	92
56	26.258	α-Curcumene	C_15_H_22_	72,773	644-30-4	99
57	26.3722	β-Selinene	C_15_H_24_	74,958	17066-67-0	99
58	26.5667	(-)-α-Cedrene	C_15_H_24_	75,004	469-61-4	93
59	26.721	β-Himachalene	C_15_H_24_	74,857	1461-03-6	99
60	27.0426	Butylated Hydroxytoluene	C_15_H_24_O	91,358	128-37-0	98
61	27.1622	Bicyclo[4.4.0]dec-1-ene, 2-isopropyl-5-methyl-9-methylene-	C_15_H_24_	74,782	150320-52-8	91
62	27.2951	β-Sesquiphellandrene	C_15_H_24_	74,824	20307-83-9	98
63	27.4642	(E)-1-Methyl-4-(6-methylhept-5-en-2-ylidene)cyclohex-1-ene	C_15_H_24_	74,778	53585-13-0	93
64	27.669	α-Muurolene	C_15_H_24_	74,668	10208-80-7	90
65	27.7362	(E)-α-bisabolene	C_15_H_24_	74,803	25532-79-0	93
66	27.8196	α-Calacorene	C_15_H_20_	71,052	21391-99-1	98
67	28.2962	Nerolidol	C_15_H_26_O	93,705	142-50-7	90
68	28.4516	Globulol	C_15_H_26_O	93,714	489-41-8	80
69	28.7264	Spathulenol	C_15_H_24_O	91,515	6750-60-3	99
70	28.8091	Caryophyllene oxide	C_15_H_24_O	91,338	1139-30-6	94
71	29.0399	Allocedrol	C_15_H_26_O	93,827	50657-30-2	94
72	29.2214	Longiborneol	C_15_H_26_O	93,880	465-24-7	99
73	29.3142	Cedrol	C_15_H_26_O	93,690	77-53-2	96
74	29.4445	Humulene epoxide ii	C_15_H_24_O	91,455	19888-34-7	99
75	29.6753	(+)-Ledene	C_15_H_24_	75,023	21747-46-6	89
76	29.8335	α-Elemene	C_15_H_24_	74,896	5951-67-7	96
77	30.1273	(-)-Spathulenol	C_15_H_24_O	91,306	77171-55-2	95
78	30.2109	T-Cadinol	C_15_H_26_O	93,720	5937-11-1	96
79	30.4515	3-Butylisobenzofuran-1(3H)-one	C_12_H_14_O_2_	60,757	6066-49-5	95
80	30.8091	β-Carotene	C_15_H_26_O	93,766	1000374-17-9	87
81	30.9389	Z-Butylidenephthalide	C_12_H_12_O_2_	59,093	72917-31-8	98
82	31.3559	(±)-Dictyopterene A	C_11_H_18_	26,814	22822-99-7	90
83	32.0237	Senkyunolide	C_12_H_16_O_2_	62,518	63038-10-8	94
84	32.1513	Neocnidilide	C_12_H_18_O_2_	64,871	4567-33-3	94
85	32.4096	(E)-Ligustilide	C_12_H_14_O_2_	60,851	81944-08-3	99
86	33.0906	Cyclopentadecane	C_15_H_30_	81,273	295-48-7	95
87	35.074	1-Formyl-2,2-dimethyl-3-trans-(3-methyl-but-2-enyl)-6-methylidene-cyclohexane	C_15_H_24_O	91,474	1000144-09-7	83
88	35.638	Rimuene	C_20_H_32_	146,645	1686-67-5	99
89	36.0701	Methyl palmitate	C_17_H_34_O_2_	144,202	112-39-0	98
90	36.2955	(-)-15-Beyerene	C_20_H_32_	146,582	2359-73-1	98
91	36.6303	m-Camphorene	C_20_H_32_	146,589	20016-73-3	99
92	36.7133	(E)-1-(6,10-Dimethylundeca-5,9-dien-2-yl)-4-methylbenzene	C_20_H_30_	144,404	55968-43-9	92
93	36.9199	n-Hexadecanoic acid	C_16_H_32_O_2_	129,145	00057-10-3	99
94	37.3025	p-Camphorene	C_20_H_32_	146,590	20016-72-2	98
95	37.393	Ethyl palmitate	C_18_H_36_O_2_	159,424	628-97-7	93
96	38.1329	Senkyunolide H	C_12_H_16_O_4_	95,394	94596-27-7	99
97	38.7307	7-Isopropyl-1,1,4a-trimethyl-1,2,3,4,4a,9,10,10a-octahydrophenanthrene	C_20_H_30_	144,408	109680-01-5	99
98	39.3893	trans-13-Octadecenoic acid, methyl ester	C_19_H_36_O_2_	172,356	1000333-61-3	97
99	39.8614	Methyl stearate	C_19_H_38_O_2_	174,782	112-61-8	97
100	40.1071	Linoleic acid	C_18_H_32_O_2_	154,669	60-33-3	99
101	40.3638	7-Pentadecyne	C_15_H_28_	79,246	22089-89-0	80
102	40.4889	Linoleic acid ethyl ester	C_20_H_36_O_2_	185,429	544-35-4	99
103	40.62	13-Tetradecen-1-ol acetate	C_16_H_32_O_3_	126,822	56221-91-1	90
104	42.0539	Monogynol	C_20_H_32_O	163,791	20107-90-8	86
105	42.2961	Monoolein	C_21_H_40_O_4_	234,104	111-03-5	83
106	42.4033	2-Heptadecenal	C_17_H_32_O	124,867	1000143-48-6	90
107	42.8272	Totarol	C_20_H_30_O	161,805	511-15-9	91
108	43.5722	Ferruginol	C_20_H_30_O	161,789	514-62-5	97
109	43.8788	2,4-dimethylbenzo[h]quinoline	C_15_H_13_N	78,098	605-67-4	87
110	47.3062	1,4-Bis(trimethylsilyl)benzene	C_12_H_22_Si_2_	93,144	13183-70-5	83
